# Prevalence of cerebrovascular accidents in patients with ulcerative colitis in a single academic health system

**DOI:** 10.1038/s41598-022-21253-w

**Published:** 2022-11-04

**Authors:** Erika Horta, Conor Burke-Smith, Bryant W. Megna, Kendall J. Nichols, Byron P. Vaughn, Rwoof Reshi, Eugenia Shmidt

**Affiliations:** 1 Department of Neurology, University of Arkansas Medical Sciences, 4301 W Markham St, Little Rock, AR 72205 USA; 2 University of Iowa Carver College of Medicine, 375 Newton Rd, Iowa City, IA 52242 USA; 3 Inflammatory Bowel Disease Program, Department of Gastroenterology and Hepatology, University of Minnesota, 516 Delaware St Se, Minneapolis, MN 55455 USA; 4 Department of Neurology, University of Minnesota, 516 Delaware St SE, Minneapolis, MN 55455 USA; 5 The University of Oklahoma Health Sciences Center, 865 Research Pkwy, Oklahoma City, OK 73104 USA; 6 Division of Gastroenterology, Hepatology, and Nutrition, University of Minnesota, 420 Delaware Street SE, MMC 36, Minneapolis, MN 55455 USA

**Keywords:** Crohn's disease, Ulcerative colitis, Stroke, Epidemiology, Risk factors

## Abstract

In general, IBD increases arteriovenous thromboembolic events, though the association between UC and cerebrovascular complications remains inconclusive. Some studies suggest young women with UC have an increased risk of cerebrovascular accidents (CVA). The focus of this study was to characterize the rates, anatomic distribution, and risk factors for CVA in patients with UC. We developed a retrospective cohort of patients with UC at a single health care system from June 2010 to June 2015. Neuroimaging was used to document presence, location and type of stroke and traditional risk factors were considered. Prevalence of CVAs in patients with UC was compared to that of the general population of Minnesota (MN) and the United States (U.S.). A total of 2,183 UC patients were identified (1088 females [f-UC], 1095 males [m-UC]). The prevalence of CVA in UC patients (4.7%, 95% CI 3.9–5.6) was higher than in the U.S. (2.5–2.7%, p < 0.0001) and in Minnesota (1.8% CI 1.5–2.2, p < 0.0001) . The prevalence increased in both sexes with a peak prevalence of 24.7% (95% CI 17.1–34.4) in women with UC over the age of 80. Older age, cancer and atrial fibrillation were risk factors for CVA in univariate analysis for both sexes. In multifactorial analysis, both age and atrial fibrillation were risk factors for CVA in the m-UC cohort, but only age was associated with CVA in f-UC. The most common type of CVA was ischemic stroke (77.7%). The most common locations for CVAs in UC patients were frontal and occipital lobes (19% and 18%, respectively). UC patients have an increased risk for CVA, with women over 80 demonstrating the highest risk. Providers should be aware of these risks in making treatment decisions for UC.

## Introduction

Ulcerative colitis (UC) and Crohn’s disease (CD) are the two most common forms of inflammatory bowel disease (IBD) and are thought to arise from an abnormal immune response to the intestinal microbiota in a genetically susceptible host^1^. IBD is considered a prothrombotic state and is associated with an increase in both venous and arterial thromboses^2–4^. This prothrombotic state is driven by a combination of acquired risk factors (e.g. corticosteroids and cigarette smoking), abnormalities of the coagulation cascade (increased fibrinogen and decreased tPA), inflammation, and endothelial dysfunction^5^. Clinical risk for thromboembolism in general is securely linked to IBD, especially in the setting of active disease. However, the drivers of cerebrovascular accidents (CVAs) in these patients remain less well understood.

IBD is associated with chronic inflammation, higher prevalence of atrial fibrillation (AF), hypercoagulability, and various vasculitides; all of which are independently related to the risk of CVA^6–8^. The literature remains incomplete regarding the universal association between IBD and CVA, with some studies suggesting an increased risk of CVA only in certain subsets of IBD patients. For example, some studies found an increased incidence of CVA in CD, while UC patients were comparatively spared^2,3,9–13^. On the other hand, specific subgroups of UC patients seem to be more affected than others, including younger women (controlling for contraception use), those with concurrent AF, and those with active inflammation^3,14^. Moreover, most of the above population-level studies investigating the association between IBD and CVA were conducted outside of the United States (U.S.) which leaves a gap in the literature regarding the distribution of IBD-associated CVAs in this country. Additionally, the typical vascular location of CVAs is not known in IBD patients. The variable CVA rates in subgroups of IBD patients are not at this time fully described or understood.

Our study analyzed primary source data to assess prevalence of CVA in a cohort of patients with UC seen in a large academic and community health care system in Minnesota (MN). We sought to define the nature of CVA in these patients (i.e. vascular localization, ischemic vs hemorrhagic, etc.—as we were able given data at hand) and to further characterize the associated risk factors.

## Methods

### Study design

This study was approved by the Institutional Review Board of the University of Minnesota (IRB#1501M59201) and underwent ethical evaluation. Informed patient consent was obtained for participation in research and all patient data was de-identified. Our work adhered to the STROBE guidelines for observational studies and was performed in accordance with the Declaration of Helsinki. See appendices for Strobe checklist. We extracted data from a Clinical Data Repository (CDR) cultivated via the University of Minnesota/Fairview Health System electronic medical record (EMR). The CDR consists of EMR data from over 2.5 million patients across 8 hospitals and 40 clinics including a large tertiary care academic center and its community affiliates. All patients who had an encounter at University of Minnesota and its affiliated clinics between June, 2010 and June 2015, with ICD 9/10 codes of 556.9/K51.xx, were eligible for inclusion in the study. Patients were excluded if their diagnosis of Inflammatory Bowel Disease (IBD) was not confirmed by a biopsy or diagnosed by a gastroenterologist or a colorectal surgeon through endoscopic features.

Demographic and clinically relevant data were extracted from the EMR. Relevant clinical information included age, sex, race, specific medical comorbidities (AF, cancer) and presence/absence of CVA. All patients with radiographic studies of the brain were identified, and these images were secondarily reviewed for evidence of CVA, specifically ischemic or hemorrhagic stroke.

Given our group’s previous findings of increased prevalence of strokes among female Ulcerative Colitis (f-UC) patients, we wanted to explore if this finding extends beyond Ulcerative Colitis (UC). We identified all female patients with Crohn’s Disease (CD) and randomly matched female patients with documented Crohn’s Disease females (f-CD) (ICD 9/10 codes, 555.9/K50.XX) by age to f-UC patients seen at the same institution within the same time period. Comparisons between f-UC and f-CD patients were also analyzed using Pearson’s test.

**Figure 1:**
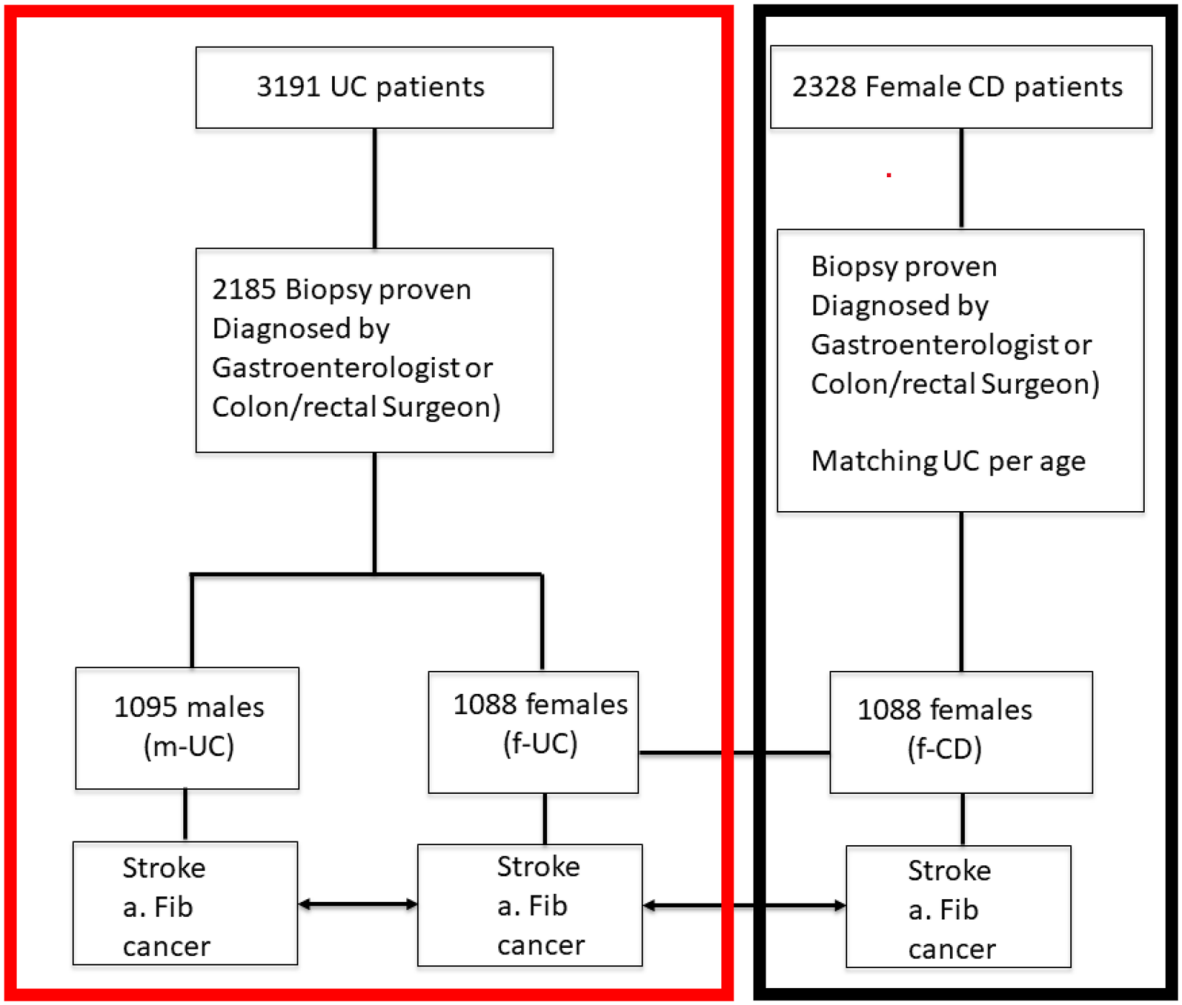
Patient selection and study design. *A. Fib* atrial fibrillation, *CD* Crohns disease, *f-CD* females with CD group, *f-UC* females with UC group, *m-UC* males with UC group, *UC* Ulcerative Colitis. (Red- Stage I) and Black (Stage 2).

Figure 1 summarizes patient selection and study design.

### Definition of patients and conditions

Diagnoses of UC and CD were established based on typical endoscopic and histologic findings consistent with IBD as interpreted by the treating gastroenterologist or colorectal surgeon. CVA was defined as the presence of hemorrhagic or ischemic stroke on neuroimaging, venous sinus thrombosis, amaurosis fugax, TIA, or history of alteplase administration during a stroke code and/or neurological deficits documented by a neurologist.

### Statistical analysis

The prevalence of CVA in the UC patient cohort was compared to the national and state-level (Minnesota-MN) prevalence of stroke reported by the American Heart Association (AHA) and the Centers for Disease Control and Prevention (CDC) over the same period of time. Patient sex, presence of AF, and cancer were treated as binomial variables and Pearson’s test was used for this analysis. Kruskal–Wallis test was used when age was treated as a continuous variable. Variables that were statistically significant on univariate analysis were analyzed via multivariate analysis to identify variables that were independently associated with CVA in patients with UC.

All statistical analyses were performed with the use of JMP software (SAS institute, version 15.2.0). When multiple comparisons were done, Bonferroni correction was used and only p-values less than 0.006 were considered significant, otherwise, p-values < 0.05 were considered significant.

### Investigation guidelines

Our work adhered to the STROBE guidelines for observational studies. See Appendices. Further, our study was performed in accordance with the Declaration of Helsinki.

### Ethics approval

The IRB of the University of Minnesota reviewed and approved this study.

## Ethical considerations

Our study was approved by the IRB of the University of Minnesota, adhered to the STROBE Guidelines, and was performed in accordance with the Declaration of Helsinki.

## Results

A total of 2183 UC patients were identified. Patient demographics, comorbidities, prevalence and location of CVAs per sex are illustrated in Table 1.

**Table 1:** Demographics and comorbidities of patients with IBD.

	f-UC N (%)			m-UC N (%)			Total N (%)	p Pearson	f-CD N (%)		
Number of patients	1088			1095			2183		1088		
**Age**								0.45			
< 20	31 (2.8)			23 (2.1)			54 (2.5)		31 (2.8)		
20–40	295 (27.1)			309 (28.2)			604 (27.7)		295 (27.1)		
40–60	388 (35.7)			369 (33.7)			757 (34.7)		388 (35.7)		
60–80	281 (25.8)			322 (29.4)			611 (27.6)		281 (25.8)		
> 80	93 (8.5)			72 (6.6)			167 (7.6)		93(8.5)		
**Race**											
White	91%			90%			90%	1.0			
Unknown	5%			5%			5%				
	Stroke (55)			Stroke(47)			282 (12.9%)	0.89	Stroke (33)		
Comorbidity	Yes	No	total	Yes	No	Total	134 (6%)	0.58	yes	No	Total
Cancer	13	124	137 (12.6)	17	128	145 (13.2)			8	128	136 (12.5)
Atrial fibrillation	10	52	62 (5.7)	19	53	72 (6.6)			7	55	62 (5.7)
CVAs*	55 (5.1)			47 (4.3)			102 (4.7)	0.4	33(3.0)		
Hemorrhagic	3 (5.5)			2 (4.3)			5 (4.9)		2 (6)		
Ischemic	43 (78.2)			35 (74.5)			78 (76.5)		30 (91.0)		
TIA	9 (16.4)			9 (19.1)			18 (17.6)		1 (3)		
SVT	0			1 (2)			1 (1.0)		0 (1.0)		
Location^a^											
Frontal	9 (20)			7 (18)			16 (19)		9 (20)		
Parietal	11 (24)			3 (8)			14 (16)		9 (20)		
Temporal	4 (9)			4 (10)			8 (9)		6 (13)		
Occipital	5 (11)			10 (25)			15 (18)		6 (13)		
BG	6 (13)			7 (18)			13 (15)		7 (15)		
Cerebellum	7 (16)			5 (13)			12 (14)		8 (17)		
Brainstem	3 (7)			4 (10)			7 (8)		1 (2)		

^a^For those patients that location of stroke was available, patients with multiple stroke locations were counted only once for prevalence, but each location was counted separately for stroke location. *CVA* cerebral vascular accident, *TIA* transient ischemic attack, *SVT* sinus venous thrombosis, *BG* Basal Ganglia.

**Figure 2:**
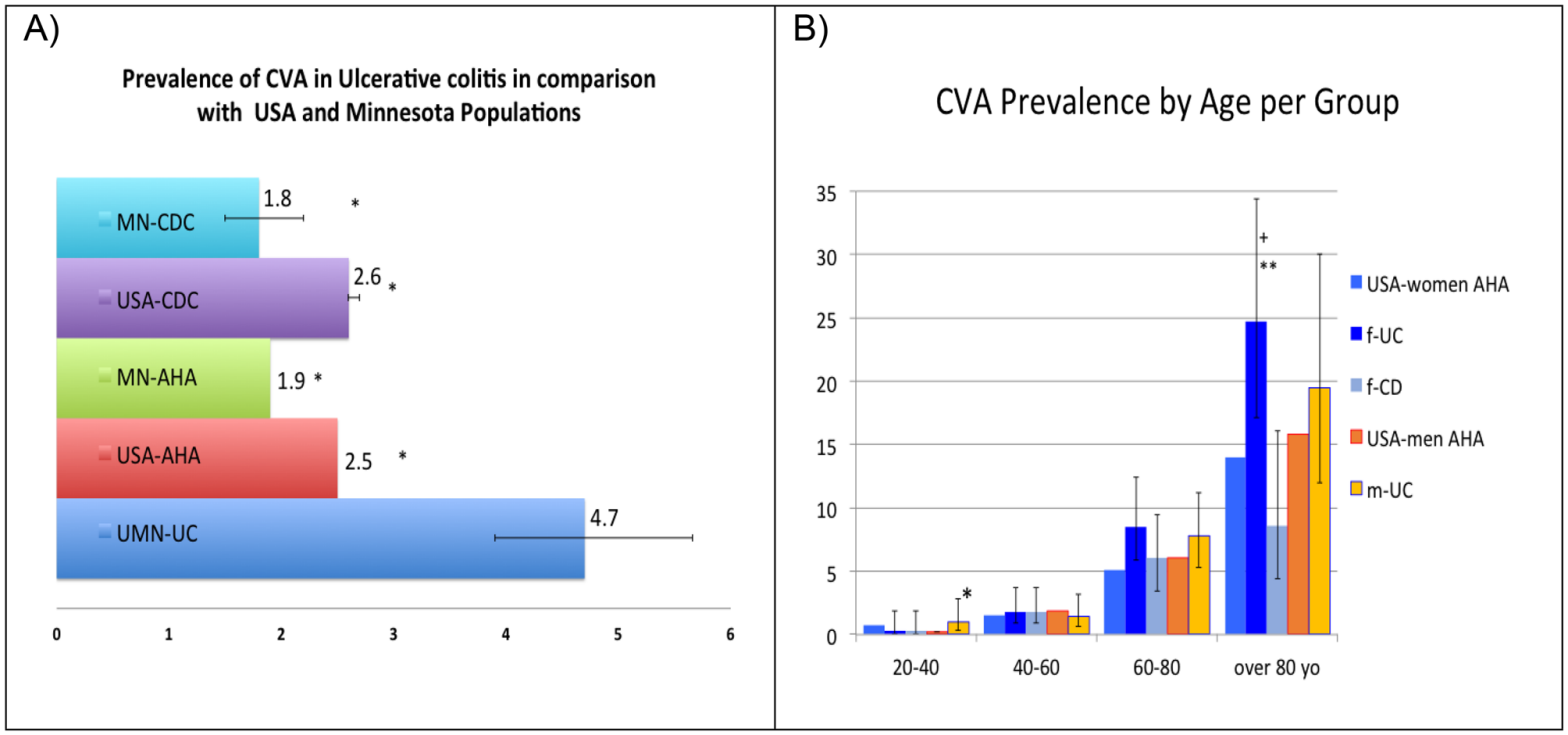
(**a**) Prevalence of cerebral vascular accidents in ulcerative colitis patients in comparison with country and state data, *p < 0.0001 (**b**) CVA prevalence by age and group, *p = 0.0024 when comparing with USA-men AHA, **p = 0.0029 when comparing with USA-women AHA, ^+^p = 0.0003 when comparing with UMN-women CD; *CVA* cerebrovascular accident, *MN* Minnesota, *CDC* Centers for Disease Control and Prevention, *AHA* American Heart Association, *UC* ulcerative colitis, *yo* years old.

Cerebral imaging was obtained in 19.9% of UC patients. The prevalence of CVA in UC patients was 4.7%, which was higher than the reported prevalence in the U.S. (per AHA and CDC) and in Minnesota. (Fig. 2a.). Prevalence of stroke in m-UC was 4.3%, (95% CI 3.2–5.7), which is higher than the CDC data for stroke in men, at 2.7% (95% CI 2.6–2.8, p = 0.0012). Prevalence of stroke in f-UC was 5.1%, (95% CI 3.9–6.5), which is greater than the CDC data for stroke in women (2.6% CI 2.5–2.7, p < 0.0001). Comparing the prevalence of CVAs per age and sex to the AHA data (Fig. 2b), f-UC over 80 years old were at disproportionately higher risk for CVA with a prevalence of 24.7% (p = 0.0029). Moreover, m-UC between 20 and 40 years old had a higher prevalence of CVA than the US population according to AHA data (p = 0.0024), but the event rate of CVA was rare with a prevalence of less than 1%. IBD subclass comparison illustrated that the prevalence of stroke in f-CD was 3.0% (vs 5.1% in f-UC, p = 0.02), and that it increased with age, though was consistently lower than f-UC across all age groups (Fig. 2b.).

Known thrombogenic risk factors for ischemic CVA, specifically cancer and AF, were investigated. The prevalence of both comorbidities was similar between f-uc and m-uc (Table 1). On univariate analysis, cancer, AF and age were statistically significant risk factors for strokes in f-UC and m-UC (Table 2). On multivariate analysis, only AF and age were risk factors for CVA among all UC patients. On multivariate analysis only age was a risk factor in f-UC while age, and AF were independently associated risk factors in m-uc (Table 2). CVA subtype is further delineated in Table 1. The anatomic localizations of strokes were not different between f-UC and m-UC.

**Table 2:** Univariate and multivariate analyses for associations between CVAs and age, atrial fibrillation and cancer per group.

	Univariate analysis			Multivariate analysis		
	Age	AF	Cancer	Age	AF	Cancer
UC	p < 0.0001 OR 0.0006–0.926	p < 0.0001 OR 4.7–12	p < 0.0001 OR 1.9–4.7	p < 0.0001 OR 10–89*	p = 0.020 OR 1.4–4.0	p = 0.66
f-UC	p < 0.0001 OR 0.0005–0.925	p < 0.0001 OR 2.0–8.8	p = 0.011 OR 1.2–4.3	p < 0.0001 OR 13.8–833* OR 7.6–50** OR 1.9–7.4***	p = 0.99	p = 0.48
m-UC	p < 0.0001 OR 0.0010–0.928	p < 0.0001 OR 6.7–24	p < 0.0001 OR 2.2–7.6	p = 0.0009 OR 0.06–0.7^ OR 0.09–0.7^^ OR 0.03–0.4* OR 0.04–0.4**	p < 0.0001 OR 2.5–10	p = 0.11
f-CD	p < 0.0001 OR 0.005–0.948	p < 0.0001 OR 2.0–12	p = 0.04 OR 1.0–5.2	p = 0.0004 OR 0.008–0.47^ OR 0.1–0.8^^ OR 0.006–0.4* OR 0.08–0.8**	p = 0.72	p = 0.11

*Comparing 20–40 years old to over 80 years old. **Comparing 40–60 years old to over 80 years old. ***Comparing 60–80 years old to over 80 years old. ^Comparing 20–40 years old to 60–80 years old ^^Comparing 40–60 years old to 60–80 years old, *AF* atrial fibrillation, *UC* ulcerative colitis, *f-UC* females with UC, *m-UC* males with UC, *f-CD* females with Crohn disease.

## Discussion

In this large, single health system, retrospective chart review, we found that CVA prevalence was higher in our cohort of UC patients than in Minnesota and the U.S. at large. The prevalence of CVA in all UC patients increased with age and the most common stroke subtype was ischemic (70%). The predominantly ischemic nature of infarction in our dataset agrees with most studies. However, the majority of stroke literature in IBD patients has presented CVAs as an issue of younger, otherwise healthy patients with severe IBD^2,3^. In contrast, our study found traditional risk factors for CVA to be common in UC. The fact that our study captured a significant degree of community (outside of tertiary center) UC cases may account for this difference.

Patterns of stroke localization are important to understand underlying pathophysiology. A relatively recent review article demonstrated that the majority of strokes identified in IBD patients were in the left or right middle cerebral artery, making anterior circulation events the most commonly affected area, similar to the general population^11,15,16^. A small series of patients with CD identified recurrent posterior circulation strokes, but the three cases described were derived from patients with recurrent strokes, which may have led to biases in the selection of patients with a predilection for this area^17^. In future studies, cerebral imaging with vascular reconstruction should be included for all patients with IBD experiencing CVAs, to better inform this issue. While it would have been ideal to classify the strokes by TOAST criteria, the data was not available in the EMR to do so. This would have further informed as to the mechanism of the strokes in IBD and should be the focus of future studies. The data we had matched the AHA and CDC data and made for perfect comparison. Current AHA and CDC data does not include TOAST classification^18^.

Stroke prevalence increased with age in IBD patients, as it also does in the overall population. The unexpected finding was the high stroke prevalence in post-menopausal women with UC. This phenomenon was even more pronounced in women over 80 years old with a much higher stroke prevalence than the overall population of the same age, and over double the number of strokes seen in elderly females with CD. The prevalence in stroke in males with UC was not significantly increased when compared with overall population. The presence of atrial fibrillation and cancer increased with age, but their prevalence was similar among males and females with UC and with female with CD, and therefore could not explain why elderly females with UC presented with increased CVAs. This finding contrasts with previous work that showed younger women with UC at greatest risk^3,14^. Specifically, Ha et al. compared women with IBD (pooled UC and CD) under age 40 to age-matched healthy controls and found young women with IBD to have a greater risk of stroke^3^. Variations in hormone levels and/or varying degrees of other cumulative sex-specific risk factors, such as hormone contraception use, may be important contributing factors to this finding. Other investigators have demonstrated that women with IBD are at increased risk for cardiovascular disease and it is presumed that hormonal variations may impart a degree of this risk^7,8,13,19^. Possible explanations are hormonal disequilibrium, inflammation, and endothelial dysfunction^20^. In this way, estrogen has myriad effects on cardiovascular health as women age and this dynamic may persist and possibly be exacerbated in the setting of IBD^21^. This is evidenced by literature supporting the role of estrogen in TNF-alpha modulation as it relates to inflammation and interactions within the gut microbiome^22^. Further, murine studies have shown that estradiol downregulates TNF-alpha and subsequently is protective against acute colitis^23^. These findings represent an intriguing avenue of investigation to further link post-menopausal women with IBD, variations in hormone levels, and increased cerebrovascular risk. Due to the retrospective nature of this investigation impact of other stroke risk factors like physical activity, BMI, and cholesterol levels were not studied. A prospective study will be needed to further elucidate these and other important risks for strokes in IBD patients.

Limitations of our study include both its retrospective nature and lack of correlative data to indicate degree of disease activity in the IBD patients at the time of CVA. Further, more generally, our data did not capture personal history of thrombophilia, tobacco smoking history, immobilization, recent surgery, use of central venous catheters, VTE prophylactic measures, IBD medications, and overall compliance. Missing data and differential loss to follow up may be products of our retrospective design. Our strict definition of stroke prioritized specificity over sensitivity and could have resulted in a lower stroke detection rate in our UC and female CD cohorts. However, an artificially low stroke detection rate should have favored the hypothesis that thromboembolism would not be increased in both cohorts, which suggests that our findings are not spurious. Further, IBD disease activity as illustrated via serum C-reactive protein (CRP) levels, fecal calprotectin levels or better yet, endoscopic impression would allow inferences to be made concerning degree of inflammation and thrombotic risk. This is important, as while quiescent disease exhibits a measurable degree of risk, active IBD flares generate the highest risk of VTE propagation, atrial fibrillation, and stroke^12,24^. Further, increased disease activity is also linked to higher risk of myocardial infarction, stroke, and cardiovascular-related death, with elevated CRP levels acting as a surrogate for disease activity^25^. Lastly, while it is important that we incorporated active cancer and atrial fibrillation, a more comprehensive co-variate model including additional known risk factors for CVA may have yielded a more comprehensive analysis.

## Conclusions

In conclusion, we have demonstrated that, compared to state and nationwide general populations, females with ulcerative colitis are at an increased risk for stroke, especially as they age beyond 80. Therefore, mitigation of risk factors for ischemic stroke, including aspirin prescription as stroke prevention, should be addressed by gastroenterologists, cardiologists and neurologists taking care of patients with UC. In our study, although cancer and atrial fibrillation were prevalent in UC patients experiencing strokes, only older age persisted as a risk factor for strokes in women with UC. Also, atrial fibrillation may exist as a contributor to strokes in men with ulcerative colitis. Further studies are needed to determine the distinct etiologies for the increased prevalence of CVA in patients with all subtypes of IBD and to further delineate sex-related risk. Ideally, these future investigations will help to design individualized primary and secondary stroke prevention strategies to improve cardiovascular outcomes in patients with IBD.
